# Land Use and West Nile Virus Seroprevalence in Wild Mammals

**DOI:** 10.3201/eid1406.070352

**Published:** 2008-06

**Authors:** Andrés Gómez, A. Marm Kilpatrick, Laura D. Kramer, Alan P. Dupuis, Joseph G. Maffei, Scott J. Goetz, Peter P. Marra, Peter Daszak, A. Alonso Aguirre

**Affiliations:** * Columbia University, New York, New York, USA; † The Consortium for Conservation Medicine, New York; ‡ University of California, Santa Cruz, California, USA; § New York State Department of Health, Slingerlands, New York; ¶ Woods Hole Research Center, Falmouth, Massachusetts, USA; # Smithsonian Environmental Research Center, Edgewater, Maryland, USA; ** National Zoological Park, Washington, DC, USA; †† Wildlife Trust, New York

**Keywords:** Land use, disease, urbanization, serology, Sciurus carolinensis, indicator species, risk, dispatch

## Abstract

We examined West Nile virus (WNV) seroprevalence in wild mammals along a forest-to-urban gradient in the US mid-Atlantic region. WNV antibody prevalence increased with age, urbanization, and date of capture for juveniles and varied significantly between species. These findings suggest several requirements for using mammals as indicators of transmission.

West Nile virus (WNV) is maintained in an enzootic bird-mosquito-bird cycle and is transmitted by numerous mosquito species, including many that feed on mammals (*1*). Several mammal species have been found to be naturally exposed to WNV, and it has been suggested that wild mammals could be used as indicators of transmission (*2**–**4*). WNV seroprevalence in wild mammals will be a useful indicator of WNV activity only if it differs between sites, if it reflects within-season transmission, and if other key confounding factors are accounted for.

To test 4 hypotheses about the exposure of mammals to WNV, we examined WNV seroprevalence in wild mammals in the eastern United States. First, we predicted that WNV seroprevalence would differ significantly among species because of differences in mosquito preferences, mammal behavior and survival, and other factors (*2**,**3*). Second, we predicted that seroprevalence would be higher for adults than for juveniles because adults have been exposed to WNV for at least 1 additional year. Third, we predicted that WNV exposure would increase with the date of capture over the transmission season because peak transmission occurs during late summer. Finally, we predicted that WNV seroprevalence would vary among sites and increase with urbanization because the abundance of *Culex pipiens*, the dominant enzootic vector in this region (*1*), increases with human population density (*4*).

## The Study

We trapped mammals at 7 sites along a forest-to-urban gradient in Maryland and Washington, DC, USA, from early June to late September 2005 and in April 2006. The sites included 1 forested area (Smithsonian Environmental Research Center, Edgewater, MD), 2 large wooded parks (Rock Creek Park, Rockville, MD; Fort Dupont Park, Washington, DC), 2 residential neighborhoods (Takoma Park, MD; Bethesda, MD), and 2 urban areas (Baltimore, MD; Washington, DC).

We quantified the land use around each site by calculating an urbanization index (UI) within a 1,000-m radius as follows:

UI = (100% – % tree cover + % impervious surface)/2

Impervious land and forest cover were estimated by using multitemporal (leaf-on and leaf-off) compilations of Landsat satellite images at 30-m spatial resolution, higher resolution satellite imagery, and digital orthophotography (*5*).

We ran trap lines of Tomahawk (models 201, 203, 204, 207; Tomahawk Live Trap Company, Tomahawk, WI, USA) and Sherman (model LFAHD; H.B. Sherman Traps, Inc., Tallahassee, FL, USA) traps for 2–5 days and nights at each site. Captured animals were chemically restrained and tagged, and age was determined by using body mass and/or reproductive characters (*6*). Blood samples (0.1 mL) were obtained, dispensed into tubes containing 0.9 mL BA-1 medium, and placed on ice packs until storage at –80°C. Blood samples were allowed to clot before antibody assays were run. We assayed the blood samples for neutralizing antibodies to WNV and Powassan virus (but not St. Louis encephalitis virus, which was absent in the local bird community at these sites [*7*]) by using the plaque-reduction neutralization test (*8*) at a 1:10 dilution, with 80% and 90% neutralization of plaques as cutoffs. We examined variation in WNV antibody prevalence by using binary logistic regression with species and age as categorical factors and capture date and urbanization index as covariates. We used October 15, 2005, as the capture date for the April 2006 samples because the abundance of WNV-infected mosquitoes falls precipitously after this date (*9*).

We obtained 244 samples from 11 mammal species (Table 1). The probability of being WNV antibody–positive varied significantly among species, was significantly higher for adults, increased with capture date for juveniles, and increased with the urbanization index (Table 2). The higher seroprevalence in samples collected in April 2006 showed that WNV exposure of juvenile eastern gray squirrels (*Sciurus carolinensis*) continued after the last trapping periods in September 2005 (Table 2).

**Table 1:** West Nile virus in wild mammals at 7 sites in Washington, DC, and Maryland, United States*

Capture site	UI	Age	% WNV seroprevalence (no. samples)					
			*Tamias striatus*	*Sciurus carolinensis*	*Didelphis virginiana*	*Peromyscus leucopus*	*Procyon lotor*	*Rattus norvegicus*
Baltimore, MD	91.2	J		0 (3)				
		A		64 (10)				50 (2)
Foggy Bottom, Washington, DC†	75.5	J		20 (11)		50 (2)		
		J‡		43 (7)				
		A		52 (23)		50 (2)		50 (6)
		A‡		100 (6)				
Fort Dupont Park, Washington, DC	38.8	J		100 (2)	20 (5)			
		A		75 (8)	60 (5)		50 (2)	
Takoma Park, MD§	50.4	J		0 (2)	71 (7)			
		J‡		50 (6)				
		A		65 (20)	50 (6)		100 (2)	
		A‡		100 (5)				
Bethesda, MD¶	41.5	J	0 (4)			100 (1)		
		A	22 (12)	67 (13)				
Rock Creek Park, Rockville, MD#	27.8	J		0 (5)		0 (1)		
		A	16 (6)	30 (20)		0 (3)	100 (3)	
SERC**	16.2	J			50 (4)	0 (11)	0 (1)	
		A		100 (1)	25 (4)	0 (6)	0 (1)	

*Mammals caught from June 14, 2005, through September 17, 2005, except where noted. WNV, West Nile virus; UI, urbanization index; A, adult; J, juvenile. †Also sampled house mouse, *Mus musculus* (1 WNV-positive adult, 1 WNV -negative juvenile). ‡Samples from April 2006. §Also sampled big brown bat, *Eptesicus fuscus* (1 WNV-negative adult), little brown bat, *Myotis lucifugus* (1 WNV-positive adult). ¶Also sampled little brown bat, *Myotis lucifugus* (1 WNV-positive adult). #Also sampled groundhog, *Marmota monax* (1 WNV-negative adult). **SERC, Smithsonian Environmental Research Center, Edgewater, MD; also sampled domestic cat (1 WNV-negative juvenile), groundhog, *Marmota monax* (1 WNV-negative adult, 1 WNV-positive adult), eastern cottontail rabbit, *Sylvilagus floridanus* (1 WNV-negative adult).

**Table 2:** Logistic regression analysis of West Nile virus seroprevalence in wild mammals*

Predictor	Coefficient	Odds ratio (95% CI)	p value
Constant	–7.52 ± 2.75		0.006
Age (adult)	7.83 ± 2.97	2,508.54 (7.48–8.4 x 10^5^)	0.008
Juvenile date of capture†	0.024 ± 0.01	1.02 (1–1.05)	0.025
Adult date of capture	–0.004 ± 0.005	1 (0.99–1.01)	0.503
UI	0.015 ± 0.008	1.02 (1.0–1.03)	0.045
Species‡			0.007
* Tamias striatus*	–1.7 ± 0.68	0.18 (0.05–0.69)	0.012
* Didelphis virginiana*	0.46 ± 0.46	1.59 (0.64–3.94)	0.32
* Peromyscus leucopus*	–1.52 ± 0.68	0.22 (0.06–0.84)	0.026
* Procyon lotor*	0.88 ± 0.77	2.41 (0.53–11)	0.26
* Rattus norvegicus*	–0.7 ± 0.75	0.5 (0.11–2.19)	0.36

*Analysis used an 80% neutralization cutoff in plaque-reduction neutralization tests (PRNTs). Date refers to Julian date (January 1 = 1) and ranged from 165 (June 14) to 285 (October 15). All effects were significant when using a 90% cutoff in PRNTs at p<0.05 except urbanization index (UI) (p = 0.10). CI, confidence interval. †Squirrel samples collected in September and April at Takoma Park, MD, and Foggy Bottom, Washington DC, were significantly different (logistic regression with age, site, and month as categorical factors; September coefficient −2.22 ± 0.85; p = 0.009). ‡Species effect χ^2^ 16.3; df 5; p = 0.007. Coefficients and odds ratios used eastern gray squirrels (*Sciurus carolinensis*) for the reference level.

Seroprevalence rates were highest (and not significantly different) in 4 peridomestic species: eastern gray squirrels, Virginia opossums (*Didelphis virginiana*), raccoons (*Procyon lotor*), and Norway rats (*Rattus norvegicus*) (Tables 1, 2). Eastern gray squirrels were 5.5× more likely than eastern chipmunks (*Tamias striatus*) to have WNV antibodies and 4.5× more likely than *Peromyscus leucopus;* both differences were significant (Table 2).

## Conclusions

Previous research on the exposure of mammals to WNV has shown patterns of antibody prevalence across several states and species (*2**,**3**,**10**–**12*). However, few studies have tested for statistical differences in the factors that influence the exposure of mammals to WNV, which thus would establish their usefulness as indicators of variation in WNV transmission. We found significant effects of age, species, site, and date of capture on WNV seroprevalence.

The increase in seroprevalence with urbanization suggests that factors that increase WNV transmission, including mosquito abundance, WNV prevalence, or feeding on mammals, are higher in more urban areas. However, increased survival of mammals in urban areas could also result in increased seroprevalence associated with urbanization.

As in other studies, we found high WNV seroprevalence in 3 common peridomestic wild mammal species (*D. virginiana*, *P. lotor*, and *S. carolinensis*) and lower seroprevalence in *T. striatus* (Table 1) (*3**,**11**,**12*). Fatal WNV infection or low mosquito exposure for *T. striatus* could account for low seroprevalence in this species in areas where other species were often exposed (Table 1) (*7**,**9**,**13*). Additionally, we found that prevalence was significantly lower in juvenile *P. leucopus* in forested areas than in urbanized areas (Table 1; 0/11 vs. 2/3; Fisher exact test p = 0.032), which might explain some of the site variability found in previous studies (*3*). Finally, we found higher seroprevalence in rats than did previous studies (*3*) (4/7 vs. 2/36 [*1*]; Fisher exact test p = 0.004), which may have been a result of sampling rats from highly urban areas.

In addition to host death and spatial variation in prevalence and vector abundance, vector feeding preferences may also contribute to the observed variability in WNV seroprevalence. Previous studies have shown that WNV vectors do feed on *S. carolinensis*, *P. lotor,* and *D. virginiana* (*14**,**15*), but these studies do not show data on host abundance, so feeding preferences cannot be determined. Similarly, at our sites several mammal species, including *S. carolinensis* and *D. virginiana*, were sources of *Cx. pipiens* blood meals (*7*). Unfortunately, sample sizes of blood meals that came from mammalian hosts were too small for determining relative preferences for different mammals, and most species (except *S. carolinensis*) are at low enough abundances that substantial numbers of blood meals would be required to estimate feeding preferences. Mosquito preferences for different mammal species is an area for future research.

In our study, the probability of having WNV antibodies increased with capture date for juveniles but not for adults (Table 2), which suggests that juveniles experience higher exposure and would be more useful for WNV monitoring. Higher exposure of juveniles may result from increased attractiveness to mosquitoes or weaker defensive behavior.

Mammals have been proposed as sentinels for human WNV risk because infection would indicate transmission outside the enzootic bird cycle (*3*). Our study demonstrates that wild mammals satisfy 2 critical requirements: spatial and temporal variability in exposure. Our results also show that to estimate current year transmission at the site of capture, using wild mammals as sentinels will require adequate samples of young animals that year or a longitudinal approach (*10*). Our finding that mammalian WNV seroprevalence appears to be more intense in urban areas suggests that per capita risk for exposure is higher in these areas.
